# Origin and Evolution of the Galilean Satellites Within the Jovian System

**DOI:** 10.1007/s11214-026-01295-6

**Published:** 2026-05-06

**Authors:** Francis Nimmo, Robin Canup, Yuri Fujii, André Izidoro, Jianghui Ji, William McKinnon, Olivier Mousis, Chris Ormel, Masahiro Ogihara, Dave Stevenson, Michel Blanc

**Affiliations:** 1 Department of Earth and Planetary Sciences, University of California Santa Cruz, 1156 High St, Santa Cruz, CA 95064 USA; 2 Solar System Science & Exploration Division, Southwest Research Institute, 1301 Walnut Street Suite 400, Boulder, 80302 CO USA; 3 Graduate School of Human and Environmental Studies, Kyoto University, Yoshida-Nihonmatsu, Sakyo, Kyoto, 606-8501 Japan; 4 Dept. Earth, Environmental and Planetary Sciences, Rice University, 6100 Main St, Houston, TX 77005 USA; 5 Purple Mountain Observatory, Chinese Academy of Sciences, No.10 Yuanhua Road, Nanjing, 210023 China; 6 Earth, Environmental and Planetary Sciences, Washington University, St. Louis, MO 63130-4899 USA; 7 Institut Origines, LAM, Aix-Marseille Université, CNRS, CNES, Marseille, France; 8 Institut Universitaire de France (IUF), Paris, France; 9 Department of Astronomy, Tsinghua University, Beijing, 100084 China; 10 Tsung-Dao Lee Institute, Shanghai Jiao Tong University, 1 Lisuo Road, Shanghai, 201210 China; 11 Division of Geological and Planetary Sciences, California Institute of Technology, 1200 E California Boulevard, Pasadena, 91125 CA USA; 12 IRAP, Universite de Toulouse, Toulouse, F-31400 France; 13 Weihai Institute for Interdisciplinary Research, Shandong University, Weihai, 264209 China

**Keywords:** Satellites, Accretion, Origins, Differentiation, Jupiter

## Abstract

We review the key observations and theories relevant to the origin and evolution of the Galilean satellites. Key observations include: the potentially undifferentiated nature of Callisto; the increasing ice fraction with semi-major axis; the present-day existence of the Laplace resonance; the potential resurfacing of Ganymede mid-way through its evolution; and the metal-enriched nature of Jupiter’s envelope. The most widely accepted theory for the formation of the satellites is the so-called “starved disk” model, although newer alternatives including decretion disks and pebble accretion have also been proposed. Models that allow slow satellite formation in a cold disk are preferred, based on the density progression and Callisto’s apparent differentiation state. Major model uncertainties include the angular momentum distribution of the material infalling to the circumplanetary disk, the source of the solids, and the thermal and viscosity structure of the disk. We identify six outstanding questions, some of which will be answered by JUICE, Europa Clipper and Tianwen-4. A major difficulty in answering some questions is overprinting of primordial characteristics by later events.

## Introduction

The Galilean satellites present a study in contrasts: Io is volcanically hyperactive, Europa has a thin ice shell overlying an ocean, Ganymede is the only moon to generate its own present-day magnetic field, and Callisto may be incompletely differentiated. However, they also display orderly behavior: there is a smooth progression in density with semi-major axis, and the inner three moons are locked in the famous 1:2:4 Laplace orbital resonance. The aim of this article is to use these (and other) observations to probe how the Galilean moons formed and evolved. Successful models would ideally also be applicable to the Uranian and Saturnian moons, and could also potentially inform our understanding of exoplanet origins and evolution.

The article begins with a discussion of the formation of Jupiter, which sets the boundary conditions (e.g. temperature, gas density) governing the origins of the moons. The composition of Jupiter may also provide a proxy for the initial composition of the moons’ building blocks. We then proceed to summarize the currently available observational constraints pertinent to moon formation and evolution. The bulk of the article is a discussion of the different accretion pathways that have been proposed, and how they may be distinguished. Key topics include the behavior of the accreting gas circumplanetary disk (CPD), the source(s) of the accreting solids, and their size distribution (pebbles or planetesimals?). This section is followed by a discussion of post-accretion evolution, including the history of the Laplace resonance. Finally, we identify key outstanding questions, breaking them down into questions likely to be answered by forthcoming missions (Europa Clipper, JUICE and Tianwen-4), and those that will require subsequent missions.

## Current Understanding of Jupiter Formation

In early work on Jupiter’s formation, two main ideas were followed. The first assumed that Jupiter formation was analogous to the formation of stars by gravitational (Jeans) instability (Toomre 1964; Goldreich and Ward 1973; Boss 2019). This approach is not currently favored and appears to have multiple problems, both for the planets in our solar system and for the vast majority of known exoplanets. The most frequently stated difficulty lies in the presence of heavy elements (everything other than H and He) at abundances (higher than solar) and spatial distributions (varying with position within the planet) that seem incompatible with the simple Jeans picture; indeed this is why Perri and Cameron (1974) abandoned it in favor of core accretion, described below. There are also theoretical concerns that center around the tendency for disks to redistribute material by gravitational torques before the needed instability is reached.

We focus instead on the widely favored and more likely scenario in which the formation of Jupiter (and perhaps giant planets in general) is accomplished by first forming a core of heavy elements, embedded within a gaseous nebula, and followed by accumulation of gas from the disk. The accretion of gas and solids is actually concurrent, but initially solids dominate, whereas later gas is accreted at a much larger rate than are solids. This gas accretion is eventually stopped by a combination of gap formation and the dispersal of the gaseous component of the proto-planetary disk (PPD), a process that is observed to happen on average around 3 Ma after the initial collapse from the interstellar medium (Haisch Jr et al. 2001; Williams and Cieza 2011). The development of a gap or barrier to the migration of solids in the proto-planetary disk is now likely thought to be expressed in the different isotopic characteristic of different classes of meteorites (Kruijer et al. 2020), though the efficacy of this barrier is debated (Szulágyi et al. 2022). Gas flow onto proto-Jupiter is expected to be nearly vertical (Tanigawa et al. 2012); the extent to which this flow is concentrated near Jupiter itself has important implications for the subsequent evolution of the disk angular momentum (see below). Because of settling of solids towards the mid-plane, this infalling gas might be depleted in solids (e.g., Shibaike et al. 2019), although the picture is currently unclear (Szulágyi et al. 2022). Understanding the availability of infalling solids is obviously of great importance to understanding satellite formation.

Even in the core accretion scenario, there was still the notion of a possible gravitational instability or equivalently a “critical” core mass (Perri and Cameron 1974; Mizuno 1980; Stevenson 1982; Bodenheimer and Pollack 1986; Wuchterl 1993), but there was no fully consistent approach to the concurrent accretion of solids and gas until the seminal work of Pollack et al. (1996). They found that the accumulation of a massive envelope was mediated by the need for the envelope to lose heat, so that opacity is a key factor (see also Venturini and Helled 2020). An important consequence of this result is the absence of a well-defined critical mass, although a core of order $10~M_{Earth}$ still remains a relevant goal, since it is still true that the core mass and elapsed time determine the ability of the gas envelope to grow. Depending on the distance, the amount of heavy elements available in the protosolar nebula may well have permitted that amount or even an order of magnitude more.

In this picture, the formation of Jupiter can be thought of (simplistically) as three stages: First, a rapid accretion (in a few hundred thousand years) of a central heavy element component (arriving as solids and initially thought of as remaining a solid since the thermal aspects were not considered in the early work). This would have largely depleted the heavy elements (presumed to be partially or mostly disconnected from any gas flow) in the radial zone centered on proto-Jupiter. Second, an aggregation of a gas envelope (though still with some solids aggregating because of the growth of the Hill sphere) that is quasi-static (i.e., hydrostatic equilibrium is close to being satisfied) but is mediated by the ability of the body to lose heat. As a consequence, this stage can last several million years and was thought by some to be a negative aspect of the model since it suggested a substantially delayed formation of Jupiter.^1^ Third, a “runaway” of gas inflow once the mass of accreted gas becomes comparable to the centralised heavy element mass component. The timescale for this is dynamical, not thermal, and may be limited only by the rate at which the planetary embryo encounters nebula gas. As a consequence of its short duration, accretion in this epoch might be heavy element-poor.

Later work in the same spirit (Stevenson et al. 2022) retains much the same features but includes two very important modifications: the vaporization of incoming solids, and the late infall of around an Earth mass of solids (possibly relevant to the formation of the Galilean satellites but motivated by the observation that the Jovian atmosphere is enriched relative to solar abundance of heavy elements by roughly a factor of three, something that is otherwise hard to explain). The unavoidable severe heating of incoming solids (Brouwers and Ormel 2020), even when the embryo is only a few Earth masses, likely nullifies the concept that the innermost region is solid,^2^ and goes part way towards explaining why it is incorrect to imagine Jupiter as a core of heavy elements surrounded by an envelope of at most mildly heavy-element enriched H-He.

The resulting diluted core is not, however, as dilute as the analysis of Juno gravity data suggests. Multiple models (e.g., Helled et al. 2022) require that the heavy element component of Jupiter be distributed outward by a large amount. Keeping in mind the cubic dependence of volume on radius but also the decrease in H-He density with radius, the dilution of an otherwise purely heavy element component of about 0.2 Jupiter radii^3^ out to, say 0.6 Jupiter radii (a value favored in the models of Militzer, for example) is an almost order of magnitude dilution of the innermost H-He mass. In short, the deepest part of Jupiter could be “dirty” H-He rather than heavy elements with some modest admixture of H-He.^4^

The cause of the extensive dilution is not well understood but a more complicated story of accretion - perhaps involving a period of slow growth with solid-enhanced material - is one possibility. In this concept, an extended stage 2 in which gas accretion is accompanied by a planetesimal accretion rate high enough to delay cooling and the onset of runaway gas accretion until the planet mass reaches $\sim 10^{2}~M_{Earth}$ yields an extended heavy element distribution consistent with the data (Helled et al. 2022). This hypothesis might have relevance to the origin of the material that comprises the Galilean satellites as well as the enrichment of the outer Jovian envelope, although whether high solid accretion rates during Jupiter’s primary growth would still apply at the very end of its gas accretion is unknown.

How might this or some similar accretion story impact our understanding of the origin and nature of the Galilean satellites? Three issues come to mind: CPD angular momentum, temperatures, and magnetic fields.

(1) *Angular Momentum and Spin*

The Jupiter accretion models discussed above are mostly spherically symmetric and therefore ignore rotation. The centripetal acceleration at Jupiter’s equator is an order of magnitude less than gravity so the internal structure is only mildly affected by its rotation, suggesting that the main elements described above are still correct. An early Jupiter can easily be twice the radius of the current Jupiter (albeit only for a timescale of order a few million years, not very different from the timescale of accretion) (Fortney et al. 2011; Batygin and Adams 2025), increasing the rotation period to of order 40 hours and reducing the role of rotation even further. However, this says nothing about the likely presence of a gas circumplanetary disk. Unlike planetesimals (which can have incoming 3D velocities distributed almost randomly around zero in the Jupiter embryo frame of reference and therefore tend to impart little spin except for the possibility of very large embryos as in the formation of Earth-Moon), the inflow of gas is thought to be coherent and prograde (i.e., in the same sense as the planet’s orbit about the Sun), and could in principle impart significant accreted angular momentum that would have to be accommodated in a disk. Compared to the present-day spin angular momentum of Jupiter (or that of the Galilean satellites), that of infalling accreting material is huge, strongly implying the existence of a substantial circumplanetary disk (CPD) (Mosqueira and Estrada 2003)

An important quantity is the centrifugal radius $r_{c}$, the distance at which the specific angular momentum of the infalling material equals that of a circular orbit around Jupiter. This distance sets the region where gas is initially delivered to the CPD, with the gas subsequently evolving both inwards and outwards due to viscous spreading (Canup and Ward 2002; Batygin and Morbidelli 2020). Because $r_{c}$ effectively determines whether a disk is primarily spreading outwards (decretion) or inwards (accretion) in the satellite formation region, it plays a major role in governing satellite growth and survival (see below).

(2) *High Temperatures*

Accretion models suggest high peak luminosities. It is easy to see why this may be so: accretion of a Jupiter mass to a sphere with a radius twice the current Jupiter radius in a time of a million years implies (by the First Law of thermodynamics) a luminosity of several times $10^{-4}$ solar. For black body radiation at that radius, a temperature of around $1500\text{ K}$ is plausible. The value might be somewhat reduced if some of the incoming gravitational kinetic energy were radiated from a disk that is easily 100 times larger than the surface of protoJupiter. This possibility is not in existing accretion models which focus rather on the effects of disk viscous heating (Canup and Ward 2002). Long ago (Pollack and Reynolds 1974) it was recognized that high temperatures arising from Jupiter’s accretion might be relevant to the absence of ice in the inner Galilean satellites, and more recent work (Bierson et al. 2023; Schneeberger and Mousis 2025) supports this. However, the disk from which the satellites presumably formed may have been optically thick and the formation conditions of these bodies may depend as much as on understanding the disk (e.g., on its viscosity and opacity) as on Jupiter itself. This example also highlights the important role of timing: when the (surviving) satellites formed relative to Jupiter’s accretion is quite uncertain but will affect the thermal environment in which formation happened.

(3) *Magnetic Field*

In the simple models, early Jupiter may have been convective, generating a much higher heat flux and entropy than now, thus providing the necessary conditions (electrical conductivity and convection) for magnetic field generation. From the proposed scaling laws of Christensen (2010) and more recent work by Hori (2021), this field could have been at the kilogauss level and might have had important consequences for the dynamics of the disk. In particular, it might have set up an inner edge of the disk, which can in turn have large consequences for satellite accretion (Sasaki et al. 2010; Batygin 2018 but cf. Takasao et al. 2022).

In summary, the formation of Jupiter and its moons are closely related. How and where gas and solids flowed onto the growing proto-planet largely determined the temperature structure and angular momentum budget of the circumplanetary disk. The mass ratio of solids to gas in the infalling material is particularly uncertain. If Jupiter’s early magnetic field was strong enough, and the inner disk temperature high enough for ionization and magnetic coupling, a cavity may have opened up in this disk, with important consequences for satellite migration.

## Summary of Satellite Observational Constraints

As will be seen below, there are a variety of scenarios for how the Galilean satellites formed. Here we will briefly summarize the physical and chemical characteristics of the moons that can in principle be used to distinguish between different accretion scenarios. Further details can be found in Nimmo et al. (2026, this collection).

### Physical Characteristics

The two most important physical characteristics are the density and the moment of inertia. The first provides constraints on the body’s bulk composition and the second on its differentiation state. An excellent review may be found in Schubert et al. (2004).

*Density* As has long been known, the Galilean satellites show a smooth decrease in bulk density with increasing semi-major axis (Table 1). This is interpreted as a change in bulk composition from Io (ice-free) to Europa (a thin hydrosphere) to Ganymede and Callisto (roughly one-half (Mueller and McKinnon 1988) ice). What is not clear is whether this density gradient is because of conditions in the CPD during accretion, or due to some process of volatile loss that happened after accretion. We discuss this issue in more detail below. Here we will note that the (probably) unaltered ≈1:1 rock:ice ratios of Ganymede and Callisto are similar to the ≈1:1-1:3 ratio expected for a solar nebula composition (Lodders 2003), if the high-density polymorphs of water-ice are taken into account. It should also be noted that we are assuming a simple two-component (ice-rock) system. If organics are present in large quantities (Reynard and Sotin 2023, cf.) then the above rock fractions will be too high.

**Table 1:** Rock mass fraction $f_{\mathrm{rock}}$ is calculated assuming rock and ice densities of 3530 and 950 $\mathrm{kg~m^{-3}}$, respectively. Note that $f_{rock}$ is substantially lower for Ganymede and Callisto if the self-compression of the ice phases (and possible internal oceans) are considered (Lupo 1982; McKinnon 1997)

Body	Radius (km)	Bulk density	$f_{rock}$
Io	1820	3530	1.0
Europa	1565	3010	0.94
Ganymede	2634	1940	0.71
Callisto	2410	1830	0.66

*Moment of Inertia* For a body responding like a fluid (in hydrostatic equilibrium), a measurement of the physical or gravitational flattening is sufficient to determine the body’s moment of inertia (MoI) via the so-called Radau-Darwin equation (Schubert et al. 2004). The MoI indicates how centrally-condensed the body is, and thus, qualitatively, how much differentiation it has undergone. Here differentiation could mean separation of ice from rock, or rock from metal (or both). The importance of differentiation is that it requires some minimum temperature to occur — solid-state differentiation is very slow unless the denser rocky components are of large-enough scale (Stevenson 1990; Nagel et al. 2004).

For a simple two-layer body, if the bulk density and MoI are both known, the structure can be determined if one layer’s density is specified. For more complicated layering, there is insufficient information to determine a unique structure. However, a phase space of allowable interior structures can be identified as a function of the number of layers and their densities.

As an example, Io’s interior structure is probably well-approximated by a silicate mantle overlying an iron or iron-sulfide core. The inferred MoI of 0.378 (Schubert et al. 2004) has been refined by close passes by the *Juno* mission, confirming that the body is in hydrostatic equilibrium (Park et al. 2025). This value can be used to deduce a core radius of 716 km and density of 7080 $\mathrm{kg~m^{-3}}$ assuming a mantle density of 3300 $\mathrm{kg~m^{-3}}$; alternatively, Io could possess a liquid iron-sulfide core of 5150 $\mathrm{kg~m^{-3}}$ as large as 950 km radius.

Based on its high density and icy surface, Europa must have differentiated into rock plus ice/water. Whether or not it has a metallic core, however, cannot be determined based on MoI observations alone (Petricca et al. 2025), especially as the hydrostatic condition upon which the MoI determination rests remains unconfirmed (Schubert et al. 2004). Reanalysis of *Galileo* gravity data (Casajus et al. 2021) implies a lower MoI than earlier estimates, implying a correspondingly lower core rock density; as such, thermal evolution theory permits but does not require a metallic core, in the absence of substantial tidal heating (Trinh et al. 2023). In contrast, Ganymede almost certainly has an iron core because it exhibits a permanent dipole magnetic field; ergo, it is fully differentiated (metal from rock from ice (Schubert et al. 2004)).

The real question-mark is Callisto. If Callisto is in hydrostatic equilibrium, then its MoI suggests that it has not completely differentiated (Schubert et al. 2004). The reason this matters is that it doesn’t take very much energy to melt ice and thus cause differentiation. Hence, if Callisto is not fully differentiated, that places quite strict bounds both on the thermal state of the CPD as Callisto was forming, and the manner in which it formed (see Sect. 4.1.1).

However, we do not *know* that Callisto is hydrostatic, and therefore cannot be sure that it is only partially differentiated (Gao and Stevenson 2013). If we knew both the equatorial and polar gravitational flattening, we could test whether it was hydrostatic or not. But because Galileo’s flybys only provided the equatorial flattening, that test is not available. As a result, making better measurements of Callisto’s gravity is a crucial requirement for any future mission there.

### Orbital Characteristics

The Galilean satellite orbits are remarkably compact, with outermost Callisto orbiting far interior to the outermost possible distance for a prograde moon, which is at several hundred Jovian radii. That no large or midsize satellites exist beyond Callisto must thus reflect something other than dynamical stability, e.g., an inner preferred region for the delivery or assembly of satellite building-blocks. The most important orbital constraint on the Galilean satellites’ origin is that the inner three occupy the 1:2:4 Laplace resonance. How this resonance was established, and how long it has been in existence, are critical and as yet unanswered questions.

The usual presumption is that tides in Jupiter drove differential orbital migration, so that Io migrated outwards fastest and entered a resonance with Europa; the pair then continued to migrate outwards until encountering Ganymede (Yoder and Peale 1981). If this story is correct, then the age of the Laplace resonance depends on the initial satellite positions and their outwards migration rates, which depend on dissipation inside Jupiter (Lainey et al. 2009).

However, during early accretion (while gas was present), the satellites are likely to have migrated inwards via so-called Type I migration. Although the rate, and even the direction, of this migration can be sensitive to the detailed structure of the CPD (e.g., Fujii and Ogihara 2020), inwards migration is the most likely outcome. If inwards migration of the innermost body was halted (e.g. by an inner disk cavity; Sasaki et al. 2010 or by other mechanisms; Fujii et al. 2017; Arakawa and Shibaike 2019) then a resonance chain would naturally have established itself (Peale and Lee 2002; Ogihara and Ida 2012). The Laplace resonance could thus be primordial. On the other hand, the fact that Ganymede apparently underwent a tidal heating event mid-way through its history (see below) suggests it may have been in a different resonance prior to entering the present-day Laplace resonance (Showman et al. 1997), in which case the Laplace resonance cannot have been primordial.

Observational constraints on the present-day migration rates of Io and Ganymede are available (Lainey et al. 2009), with the latter moving outwards at about 11 cm/yr. The migration of Callisto has not been measured but may be comparable (Dbouk and Wisdom 2023). Unfortunately, determining past migration rates from these present-day values is very difficult as it involves the poorly-understood mechanism(s) for dissipation in Jupiter (Nimmo et al. 2023, cf.).

### Geological Characteristics

As well as density, geological activity shows a clear gradient with semi-major axis. Io is famously volcanic, with no identified impact craters to date, and Europa has a young (∼40-90 Myr old) surface (Bierhaus et al. 2009), so that neither contains any record of early solar system processes. Callisto is heavily cratered and has almost no endogenic tectonic features; it likely contains a subsurface ocean (Zimmer et al. 2000; Cochrane et al. 2025) but does not appear to ever have experienced significant deformation. From an Origins perspective, Callisto is attractive because it appears to have undergone very little post-accretion processing which might have erased the original signatures left by accretion.

Ganymede is the most interesting case, consisting of ancient (dark) and more recent (bright) terrain. Surface ages are based on poorly-known cratering fluxes (Zahnle et al. 2003; Bottke et al. 2024) and are subject to large uncertainties. Nonetheless, although the dark terrain on Ganymede is about as old as the solar system, the bright terrain is perhaps only ∼2 Gyr old with a substantial range (Pappalardo et al. 2004). This partial resurfacing event, which is widely thought to have been caused by tidal heating, provides a potential constraint on the long-term evolution of the system, and has been used to argue for resonances prior to the current Laplace resonance (Showman et al. 1997).

### Compositional Characteristics

Apart from the inferred gradient in ice fraction (Table 1), there are few available compositional constraints relevant to satellite formation. The most obvious measurements to make are of surface compositions, but these do not necessarily represent the bulk chemistry of the body and have likely been heavily processed, either inside the body (e.g., by hydrothermal activity) or at the surface (by energetic particle bombardment). Pollution by either Io or recently-infalling (asteroidal, cometary, or irregular satellite) dust is another potential concern. Measurements of interior compositions, such as those available from the plumes of Enceladus, are not currently available at the Galilean satellites.

Europa’s surface contains a range of non-ice compounds, including $\mathrm{H_{2}O_{2}}$, $\mathrm{CO_{2}}$, sulphur compounds, hydrated species and (perhaps) organics and/or clays (Carlson et al. 2009). The sulphur compounds may be from Io; most of the other species are either insufficiently certain or insufficiently diagnostic to provide useful constraints on formation processes. At Ganymede and Callisto, even less information is available.

Sodium and potassium have been observed in Europa’s atmosphere/exosphere (e.g., Brown 2001), but in a different ratio than seen near Io and in the Io torus. This hints at an intrinsic source of alkalis from Europa, but fractionation effects preclude a definite assessment (Carlson et al. 2009). Visible and ultraviolet color absorptions consistent with radiation-damaged NaCl salt are seen on Europa in Hubble Space Telescope data co-located with areas of geologically recent disruption (so-called chaos terrains) (Trumbo et al. 2022). This strongly hints at an endogenic source for the salt, presumably Europa’s ocean. James Webb Space Telescope (JWST) spectral imaging data similarly indicate $\mathrm{CO_{2}}$ ice over the same geographic area, plausibly indicating that Europa’s ocean is $\mathrm{CO_{2}}$- or carbonate bearing (Trumbo and Brown 2023; Villanueva et al. 2023).

Isotopes are potentially even more diagnostic of origin. The deuterium–to–hydrogen ratio is potentially indicative of original provenance (Horner et al. 2008; Waite et al. 2009) or evaporative loss (Bierson and Nimmo 2020; Bierson et al. 2023) and can be measured remotely in ices (Clark et al. 2019; Brown et al. 2025). Currently, the only available measurements for the Galilean moons come from the Galileo Near-Infrared Mapping Spectrometer (NIMS) data, which suggest a supersolar deuterium abundance on Callisto, albeit with significant uncertainty due to large error bars (Clark et al. 2019). The measured value is (6 ± 5) × 10^−4^, which is in agreement with the D/H ratio measured at Enceladus by the Cassini Ion and Neutral Mass Spectrometer (∼ 3 × 10^−4^; Waite et al. 2009). This consistency implies that the building blocks of Callisto may have originated from the protosolar nebula and were likely not altered by thermal heating during migration within the circumplanetary disk or by their accretion onto the growing moon. Villanueva et al. (2023) measured carbon isotope ratios in $\mathrm{CO_{2}}$ ice on Europa’s surface and deduced that the carbon could have been ultimately sourced from either $\mathrm{CO_{2}}$ ice or organic matter in the protojovian nebula, described below. Cartwright et al. (2024) have detected both ^12^C and ^13^C in solid-state $\mathrm{CO_{2}}$ on Callisto, but interpret the variations in terms of present-day radiolytic processes.

One important constraint is that the exosphere of Io is very enriched in the heavy isotopes of both sulphur and chlorine (de Kleer et al. 2024). This enrichment is likely the result of a distillation cycle whereby light isotopes are preferentially lost to outer space each time these elements are erupted. The enrichment suggests that Io’s volcanic activity has operated at levels comparable to the present day over several Gyr, thereby arguing for a long-lived Laplace resonance (or some earlier equivalent).

## Accretion Processes

Here we summarize the various processes which have been proposed as relevant to satellite accretion. We start with a discussion of generic processes within a circumplanetary disk, and then proceed to discuss specific scenarios: the minimum-mass proto-satellite nebula, the so-called “starved disk”, the decretion disk, pebble accretion and the possible role of heliocentric impactors. An excellent recent discussion of these topics may be found in McKinnon (2023), Blanc et al. (2025) is also useful, though focused mainly on the Saturnian satellites.

### Formation and Dynamics of a Pre-Satellite Disk

A giant planet contracts within its Hill sphere once the rate of its gas accretion can no longer compensate for the increasing rate of its gravitational contraction due to the planet’s growing mass and luminosity. As the planet contracts, its rotation rate increases and eventually reaches the rotational instability limit; at this point, its outer layers begin to be shed into an initial “spin-out” disk (Ward and Canup 2010). Assuming the solar nebula is still present, as contraction proceeds the planet’s radius eventually falls below the outer radius of the pattern of inflowing gas. At this point, nebular material – including both gas and entrained small solids – flows directly into orbit about the planet, and the disk is supplied both by spin-out from the planet and by inflow from the nebula (Ward and Canup 2010). During the final stages of contraction, models suggest that the disk transitions to an accretion disk supplied solely by the nebular inflow (Ward and Canup 2010).

An inflow-supplied circumplanetary disk shares basic traits with circumstellar accretion disks, within which angular momentum transport appears required to explain observed mass accretion rates onto young stars. The classic model of Lynden-Bell and Pringle (1974) describes the evolution of a gas disk’s radial surface density, $\sigma _{g}(r)$ due to a viscosity, ν, which drives angular momentum exchange between adjacent disk regions having differing orbital velocities due to Keplerian shear. The viscous shear produces a torque, $g = 3\pi \sigma _{g} \nu j$, where $j = (GM_{p}r)^{1/2}$ is the specific orbital angular momentum of a circular orbit at radius r around a planet of mass $M_{p}$. The viscous torque causes the disk to spread radially on a timescale $\tau _{\nu} \sim r^{2}/\nu $, and associated dissipation heats the disk, with a rate per disk area $\dot{E}_{\nu }\sim (9/4)\nu \Omega ^{2}\sigma _{g}$, where $\Omega \equiv (GM_{p}/r^{3})^{1/2}$ is the disk orbital frequency.

Consider a gas inflow of rate F across the disk from the planet’s surface to an outer distance set by the centrifugal radius, $r_{c}$, and a disk outer edge $r_{d} \gg r_{c}$, at which material is removed from circumplanetary orbit due to solar interactions. For a compact CPD, $\tau _{\nu}$ is only $\sim 10-10^{4}$ yr for plausible viscosity parameters (see eqn. (2) below), while F likely changes more slowly, e.g., if inflow rate is regulated by nebular dispersal occurring over $\sim 10^{6}$ yr. In this case, the gas surface density achieves a quasi-steady state that reflects a balance between the inflow supply and removal as gas spreads onto the planet or beyond $r_{d}$. For an inflow flux that is uniform with r, the resulting steady state gas surface density is a function of F, ν, $r_{c}$ and $r_{d}$ (Canup and Ward 2002), with

1 $$ \sigma _{g}(r) \approx {\frac{4F}{15\pi \nu}} f(r,r_{c},r_{d}) $$

where $f = 5/4 - (r_{c}/r_{d})^{1/2} - (r/r_{c})^{2}/4$ for $r < r_{c}$, and $f = (r_{c}/r)^{1/2}-(r_{c}/r_{d})^{1/2}$ for $r \ge r_{c}$. The gas surface density is proportional to the mass inflow rate, implying a more massive disk during rapid inflow and a decreasing $\sigma _{g}$ as the inflow wanes. The gas surface density is inversely proportional to ν because as the viscosity increases, the disk spreads more rapidly, which reduces $\sigma _{g}$ for a fixed inflow rate.

The outer edge of the inflow region, $r_{c}$, would be set by the maximum specific angular momentum of inflowing gas, which in turn depends on how Jupiter is growing (Sect. 2). If the planet accretes all material that passes within some distance from its center, and the inflowing gas has approximately Keplerian orbits about the Sun, the net angular momentum delivered by inflowing material can be simply estimated by integrating over the accreting region. Assuming this distance is the planet’s Hill radius, $R_{H} \equiv a_{p} (M_{P}/3M_{*})^{1/3}$ ($M_{p}$ and $M_{*}$ are the planet and stellar masses), and that inflow occurs primarily within the planet’s orbital plane, gives an average inflow specific angular momentum $j_{in} = lR_{H}^{2}\Omega _{p}$ (where $\Omega _{p}$ is the planet’s orbital frequency) with l=1/4 (e.g., Ruskol 1982). Once in circumplanetary orbit, such material achieves centrifugal force balance at a radius $r_{in}$ given by $(GM_{p}r_{in})^{1/2} \sim l R_{H}^{2}\Omega _{p}$, with $r_{in} = l^{2} R_{H}/3 \approx R_{H}/48$ for l=1/4. Inflowing parcels would presumably have a distribution of j about such an average. It is notable that this simple estimate gives $r_{in} \sim 15R_{J}$, comparable in radial scale to the Galilean system. However, the appropriate value of $r_{c}$ as the satellites formed remains uncertain, and models have considered a broad range of $5 \le (r_{c}/R_{J}) \le 10^{2}$ (e.g., Ward and Canup 2010, their Fig. 3; Batygin and Morbidelli 2020).

The reason that $r_{c}$ is so important is that it determines how much of the gas CPD experiences outwards as opposed to inwards spreading in the region of the accreting satellites. A small value of $r_{c}$ implies that outwards spreading dominates, leading to a so-called decretion disk. In this situation, small solids can pile up at particular distances, promoting local gravitational collapse to yield “satellitesimals” and subsequent satellite growth (Batygin and Morbidelli 2020). Conversely, in an accretion disk the inwards drift of solid material due to gas drag is accelerated by the additional inward advective motion of the gas itself, likely leading to different satellite growth pathways. In either case, the outer edge of the gas CPD is expected to be much larger than $r_{c}$ due to its viscous expansion, with an outer edge set by the distance at which disk material is stripped due to, e.g., solar torques (Canup and Ward 2002).

The most uncertain parameter in eqn. (1) is the gas viscosity ν (see Lesur et al. 2023 for a review). A simple model (Shakura and Sunyaev 1973) is often adopted with $\nu = \alpha c H \approx \alpha c^{2} \Omega $, where α is a constant, c is the gas sound speed, and $H \approx c/\Omega $ is the gas vertical scale height. There are several potential sources of viscosity. Magnetorotational instability (MRI) in weakly magnetized disks drives turbulence and outward angular momentum transport (Balbus and Hawley 1991). MRI requires a minimum ionization fraction, which can be produced thermally for temperatures in excess of $\sim 10^{3}\text{ K}$ (Keith and Wardle 2014; Fujii et al. 2017). However, the presence of grains can deactivate MRI because they are effective charge absorbers (Fujii et al. 2011, 2014; Turner et al. 2014; Keith and Wardle 2014). Other proposed sources of viscosity include vortices (e.g., Bae et al. 2015) and the shock front at the interface between infalling mass and the orbiting disk (Cassen and Moosman 1981; Lesur et al. 2015). It is also possible that the effective CPD α value varies with height, e.g., with strong angular momentum transport at altitude surrounding a more quiescent mid-plane (Shibaike and Mori 2023). The range of effective α that could be produced by such mechanisms is large, $10^{-4} \le \alpha \le 0.1$. Across this range, the implied disk viscous spreading time is short compared to the timescale for giant planet gas accretion (perhaps $10^{5}$ to $10^{6}$ yr), with

2 $$ \tau _{\nu} \sim {\frac{r^{2}}{\nu}} \sim 10^{3} \text{ yr} \left ({\frac{10^{-3}}{\alpha}}\right )\left ({\frac{r}{30R_{J}}}\right )^{3/2}\left ({\frac{0.1}{c/r\Omega}}\right )^{2}. $$

(Note that while Jupiter may rapidly open a gap in the circumstellar disk, gas is still accreted to the planet even after gap opening at a rate controlled by the circumstellar disk viscosity so long as the nebula is present (e.g., Lubow et al. 1999).)

Rock and ice needed to yield satellites must be supplied to the disk in addition to the gas, and the rate of solid delivery to the disk will likely regulate the overall growth rate of the satellites (Canup and Ward 2002). Possible sources of disk solids include 1) aerodynamic transport of small particles by the inflowing gas, 2) capture of Sun-orbiting bodies due to gas drag as they pass through the CPD, and/or 3) capture of collisional debris from collisions between planetesimals within the planet’s Hill sphere, or between a planetesimal and a planet-orbiting object. Mechanism (1) would deliver only small, ≪ meter-scale pebbles or grains to the $r\le r_{c}$ region (Canup and Ward 2002), while mechanisms (2) (e.g., Shibaike et al. 2019; Ronnet and Johansen 2020; Madeira et al. 2021; Mosqueira et al. 2010) or (3) (e.g., Estrada and Mosqueira 2006) could supply larger bodies across the whole disk for $r < r_{d}$. The relative contributions of these mechanisms remains uncertain due to uncertainty in the size distribution of small material in the circumsolar disk at the time the satellites formed, as well as dynamics of grains in the vicinity of a nearly fully formed Jupiter (e.g., Szulágyi et al. 2022).

Accreting solids would be strongly affected by interactions with the circumplanetary gas disk, through processes that have been extensively studied in the context of planet accretion within circumstellar disks. Initially small grains are highly coupled to the gas and evolve with it. As pebbles and larger bodies form, they increasingly decouple from the gas and undergo aerodynamic drag due to the difference between their orbital velocity and that of the pressure supported gas, leading to inward particle drift (for gas pressures that decline with radial distance) (Canup and Ward 2002; Shibaike et al. 2017). Formation of ≫ km-scale “satellitesimals” requires concentration of solids sufficient for local gravitational collapse due to, e.g., the streaming instability (Johansen and Youdin 2007; Drążkowska and Szulágyi 2018), accretionary growth if relative velocities are small enough to avoid collisional fragmentation, and/or capture of satellitesimals from circumsolar orbit.

Satellitesimal collisions lead to gravitationally bound mergers and the accretion of satellites. Large satellites are subject to inward Type I migration, due to the net torque on a satellite’s orbit caused by the pattern of spiral density waves in the gas disk induced by the satellite’s gravity. The fiducial Type I migration timescale for a mass M satellite is

3 $$ \tau _{I} \sim {\frac{1}{C_{a}}}\left ( {\frac{M_{p}}{M}}\right ) \left ({\frac{M_{p}}{r^{2}\sigma _{g}}}\right ) \left ({\frac{c}{r \Omega}}\right ), $$

where $C_{a}$ is a torque constant of order unity. Because the density wave torque is proportional to $M^{2}$, while the satellite’s orbital angular momentum is proportional to M, inward Type I migration becomes more rapid as a satellite grows (e.g., Ward 1997; Canup and Ward 2002).

Survival of Galilean-sized moons against Type I migration is a key issue, and most models predict substantial inward migration during accretion and even multiple generations of satellites (e.g., Canup and Ward 2006; Shibaike et al. 2019; Batygin and Morbidelli 2020), depending on whether an inner disk cavity develops (see below). As satellites migrate inward, converging orbits can lead to capture into mutual mean-motion resonances, which has been explored as a potential mechanism for producing the current Laplace relation (e.g., Peale and Lee 2002; Ogihara and Ida 2012) as an alternative to the classic models that produce the Laplace configuration via later differential outward tidal migration (e.g., Yoder and Peale 1981).

An important consequence of satellitesimal collisions is that they tend to be stochastic and thus produce radial mixing which reduces or eliminates any initial compositional gradients. Dwyer et al. (2013) found that it was very hard to retain such an initial gradient during Galilean satellite growth, although the existence of “resonance chains” helped. If satellites grow predominantly by accreting pebbles, however, compositional gradients can be maintained, as pebbles are small enough to be sensitive to local thermal conditions (e.g., Ronnet and Johansen 2020), discussed below.

Inward migration of accreting satellites may be affected by the presence of a cavity in the disk near the planet. For certain cavity edge profiles, the negative Type I torque near the edge may be countered by the positive torque due to interactions with disk material orbiting within the satellite’s co-orbiting region, and the cavity can act as a migration-stopping mechanism (Sasaki et al. 2010; Ogihara and Ida 2012). An inner cavity may result via magnetic interactions between the planet and the disk, which can provide an explanation for why the gas giant planets rotate at much less than the critical rotational rate for stability that would result from a pure accretion disk scenario (e.g., Takata and Stevenson 1996).

**Fig. 1:**
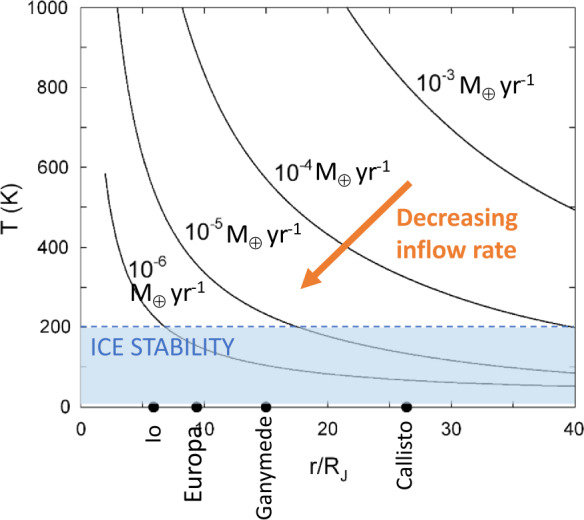
Midplane disk temperature as a function of inflow rate. Modified from Canup and Ward (2009)

An important quantity is the disk’s radial temperature profile, which affects the composition of accreting material as well as the disk’s viscosity in the alpha model. The disk is heated (with respect to the background protosolar nebular temperature) by viscous dissipation, inflow shocks, and the planet’s luminosity; these energy sources are balanced by radiative cooling from the disk surfaces. In the limit that viscous dissipation is the primary heat source (as is typically true in the regular satellite region, Canup and Ward 2002), and assuming an optically thick disk, setting the radiative cooling rate per disk area ($\dot{E}_{\mathrm{rad}}\sim \sigma _{SB}T_{\mathrm{eff}}^{4}$, where $T_{\mathrm{eff}}$ is the disk’s photospheric temperature) equal to $\dot{E}_{\nu}$ gives

4 $$ T_{\mathrm{eff}}\sim {\frac{9\Omega ^{2}}{8\sigma _{SB}}} \nu \sigma _{g} \sim {\frac{3\Omega ^{2}F}{8\pi \sigma _{SB}}}f(r,r_{c},r_{d}), $$

where we adopt the quasi-steady state expression for the gas surface density from eqn. (1) for an inflow-supplied disk. The mid-plane disk temperature, T, is related to $T_{\mathrm{eff}}$ as

5 $$ T^{4} \sim T_{\mathrm{eff}}^{4} \left ( {1 + {\frac{3}{8}} \kappa _{R} \sigma _{g} }\right ), $$

where $\kappa _{R}$ is the disk opacity. Notably, the disk effective temperature is independent of ν and $\sigma _{g}$, and is primarily a function of the inflow rate, F. Because $\sigma _{g} \propto F/\nu \propto F/(\alpha T)$, the mid-plane temperature T is proportional to $(F\kappa _{R}/\alpha )^{1/5}T_{\mathrm{eff}} \propto F^{9/20}(\kappa _{R}/ \alpha )^{1/5}$, so that it depends weakly on the opacity and viscosity and is also more strongly affected by the inflow rate. The implication is that hotter disk temperatures would apply during early rapid inflow, while the disk cools as the gas inflow rate slows. A limit on how fast the inflow could have been as the ice-rich Jovian satellites accreted can then be determined as a function of disk and infall properties (Canup and Ward 2002), and the location of the ice condensation line in the disk will move inward with time as the inflow slows. Figure 1 shows the resulting disk temperature structure for different infall rates. It demonstrates that for ice to be locally stable at Callisto and Ganymede, infall rates must have been less than 10 Earth masses per Myr. Note that inwards migration of CPD material would weaken this constraint, as long as migrating bodies were large enough not to thermally re-equilibrate before being accreted.

#### Thermal Consequences of Accretion

Accretion involves the conversion of potential and kinetic energy to heat. The warming associated with accretion can lead to differentiation, which is a key observable (Sect. 3.1). It is therefore important to understand the circumstances under which accretion can lead to high temperatures.

In order to calculate the resulting temperature, an important uncertainty turns out to be the assumed size distribution of the impactors delivered to the growing embryos. The size distribution is typically described by a power law:

6 $$ \frac{dN}{dr_{\mathrm{imp}}} \propto r_{\mathrm{imp}}^{-\alpha}, $$

with the slope α ranging from 1 to 6 (Squyres et al. 1988). The radius of impactors, $r_{\mathrm{imp}}$, can vary from a few centimeters to several hundred kilometers, depending on the disk model and the formation mechanism considered (Ronnet et al. 2017; Estrada et al. 2009; Batygin and Morbidelli 2020). Small impactors (≤100 m) are assumed to deposit energy at the radiating surface, as they are too small to penetrate deeply (Barr and Canup 2008). Medium-sized impactors (∼1 km) transfer some energy to the subsurface, allowing for conductive cooling before a new layer accumulates (Squyres et al. 1988). Large impactors (∼1–100 km), on the other hand, generate significant shock waves that can penetrate deeply into the icy-rocky structure of protosatellites (Monteux et al. 2014). The size-frequency distribution of impactors can therefore exert a strong control on the resulting satellite temperature structure (Bennacer et al. 2025).

We may consider the two end-member cases of accretion dominated by large impacts and accretion dominated by very small imapacts (“pebbles”) (Stevenson et al. 1986), recognizing that some models include both (Madeira et al. 2021; Bennacer et al. 2025). In the first case, the mean temperature increase is given by

7 $$ \Delta T \approx \frac{4\pi}{3} \frac{f G R^{2} \rho}{C_{p}} \approx 1100~\mathrm{K} \left (\frac{R}{2000~\mathrm{km}}\right )^{2} \left (\frac{\rho}{2~\mathrm{g\,cm^{-3}}} \right ) \left (\frac{f}{1}\right ). $$

Here $C_{p}$ is the specific heat capacity, R the body radius and f the fraction of impact energy that is retained as heat. For large impacts f approaches 1. Although this is a very simplified approach, Eq. (7) implies that avoiding melting and complete differentiation of Callisto-sized bodies is difficult if they are accreted from large bodies. Conversely, melting of an iron core (which at a minimum requires the Fe-FeS eutectic temperature of 1250 K) is unlikely except for the largest objects.

If accretion is dominated by small impacts, then the heat brought in to the surface is re-radiated to space. If the background (disk) temperature is $T_{0}$ and encounter velocities are neglected, then assuming an optically-thin atmosphere the final surface temperature is

8 $$ T \approx T_{0} \left [1 + \frac{4\pi}{9} \frac{G R^{3} \rho ^{2}}{\sigma T_{0}^{4} \tau}\right ]^{1/4}. $$

**Fig. 2:**
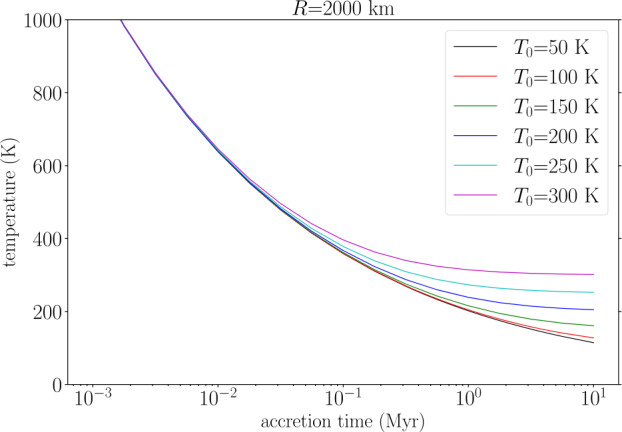
Final surface temperature as a function of accretion time and background temperature $T_{0}$ for a 2000 km radius body. Here we use Eq. (8) and take the bulk density to be 2000 $\mathrm{kg~m^{-3}}$. Pure water ice melts at 273 K at 1 bar and about 250 K at 0.2 GPa (Durham et al. 1988)

Here σ is the Stefan-Boltzmann constant and τ is the accretion timescale. (Note that aerodynamic drag of pebbles will reduce their impact energy and thus the surface temperature gain (Shibaike 2025)). With a background temperature of 100 K, accretion of a 2000 km radius body in 0.1 Myr or 1 Myr results in a surface temperature of 359 K and 204 K, respectively. As Fig. 2 shows, pebble accretion of Callisto-sized objects will result in ice melting and differentiation unless accretion timescales are long (>0.3 Myr). Accretion while radiogenic $\mathrm{^{26}Al}$ was still live would further exacerbate these problems and thus also favours longer accretion timescales (Barr and Canup 2008; Shibaike et al. 2019). Long-term heat sources such as radiogenic heating or serpentinization are likely to have smaller effects but would make avoiding differentiation more challenging. These heat sources also result in temperatures increasing with depth, whereas accretion heating can lead to a temperature profile that is (initially) reversed (Squyres et al. 1988).

To summarize, *if* Callisto is partially-differentiated (Sect. 3.1), it favours accretion scenarios with slow growth and/or small impactors (e.g., Canup and Ward 2002; Barr and Canup 2008; Shibaike et al. 2019). The fact that Ganymede has differentiated while Callisto has not is likely due to tidal heating on Ganymede (Sect. 3.3); if not, the difference might be due to late-stage impacts (Barr and Canup 2010) or variations in disk conditions (Bennacer et al. 2025) or accretion rate. The latter is not favoured, however, because of the weak dependence of temperature increase on τ in Eq. (8).

Finally, a combination of high background (disk) temperatures and temperature gains associated with either pebble accretion or giant impacts could drive volatile loss via hydrodynamic escape during accretion (Bierson and Nimmo 2020). In principle this effect could explain the observed radial density gradient; it would also predict that Europa, having lost much of its water, would have an excess of the heavy isotopes of H and O relative to Ganymede and Callisto.

### Minimum Mass Proto-Satellite Nebula

The prior section describes the formation of and key processes within a circumjovian disk produced by late gas inflow to Jupiter. But what were the disk conditions as the Galilean satellites formed? The “minimum mass subnebula” (MMSN) construct first developed in the 1980s assumes that the satellites formed from a disk having a total mass in solids (rock and ices) needed to yield the observed satellites, augmented to bulk solar composition. The MMSN disk is then gas-rich, with a $f_{g} \sim 10^{2}$ gas-to-solids ratio, a disk mass of $\sim 0.02M_{J}$ in the satellite region, and a high gas surface density, $\sigma _{g,\mathrm{MMSN}} \sim 0.02M_{J}/[\pi (30R_{J})^{2}]\sim $ few $\times 10^{5}\text{ g}/\mathrm{cm}^{2}$, where $R_{J}$ is Jupiter’s radius. For comparison, typical minimum mass solar nebula gas surface densities at 1 AU were smaller, of order $\mathrm{10^{3}~g}/\mathrm{cm^{2}}$.

An MMSN disk with a fixed total mass reflects an implicit assumption that satellite accretion commenced after the gas inflow that is thought to have produced the disk ended. This assumption is suspect, because the timescale over which an inflow-supplied circumplanetary disk forms (perhaps $10^{5}$ to $10^{6}$ yr) is much longer than the timescale for satellites to accrete from an MMSN-type disk, which is $\tau _{acc} \sim \rho _{s}R_{s}f_{g}/(\sigma _{g,\mathrm{MMSN}}F_{g}\Omega ) \ll 10^{4}$ yr for Galilean-like satellite bulk densities ($\rho _{s}$) and radii ($R_{s}$), and a gravitational focusing factor $F_{g} \ge 1$. It is thus difficult to understand why satellite accretion would “wait” until after inflow had ended. Further, the timescale to form a Callisto-sized body from a MMSN-type disk is so short that the energy of accretion would have led to complete melting of ice as Ganymede and Callisto formed, implying rapid and irreversible differentiation of rock from ice in these moons (e.g., Stevenson et al. 1986), unless there is a way to efficiently remove the heat of accretion (Lunine and Stevenson 1982). While this is consistent with Ganymede’s fully differentiated interior, it would not be consistent with an incompletely differentiated Callisto (e.g., Canup and Ward 2002; Barr and Canup 2008) (see Sect. 3.1 for a discussion of Callisto’s uncertain internal structure).

Another challenge is that the lifetime of a Galilean-sized moon in a gas-rich MMSN disk against inward Type I migration is extremely short, of order only $10^{2}$ yr (Canup and Ward 2002), so that a very rapid gas disk dispersal is needed to avoid satellite loss to inward orbital decay. A final issue is that temperatures in a MMSN disk are generally too hot for ices if the gas was at all viscous. Per the expressions above, the balance between radiative cooling and viscous dissipation in a disk with $\sigma _{g,\mathrm{MMSN}} \sim 10^{5}\text{ g}/\mathrm{cm}^{2}$ leads to photospheric temperatures in excess of $230\text{ K}$ in the region of ice-rich Callisto and Ganymede unless the viscosity was extremely low, with $\alpha \ll 10^{-4}$.

The MMSN disk was modeled after the minimum mass nebula approach for constructing the conditions in the protoplanetary disk, and was a natural starting condition for early gas giant satellite formation models (e.g., Lunine and Stevenson 1982). Several works have developed modern-MMSN type models, including the “solid enhanced minimum mass” SEMM model of Mosqueira and Estrada (2003) specifically tailored to address the above issues. However, satellite accretion models adopting such conditions have been generally unsuccessful in producing Galilean-like satellite systems (Miguel and Ida 2016). MMSN-type models are now not generally favored, with most works instead considering CPD formation via inflow and resulting satellite accretion histories for a variety of inflow and solid delivery conditions.

### Starved Disk and Its Variants

In contrast to the MMSN, where all the material making up the satellites is present at the same time, the “starved disk” model (Canup and Ward 2002) assumes material is progressively supplied to the disk at a steady rate. Because the disk undergoes viscous spreading at the same time as material is being added, an equilibrium surface density profile results, with higher inflow rates or lower viscosities (lower spreading rates) yielding higher surface densities. These surface densities can be much lower than in the MMSN case.

The starved disk scenario has multiple advantages in terms of satellite formation. First, for sufficiently slow inflow rates, the temperatures in the disk are compatible with the survival of ice-rich bodies (Fig. 1). Second, the resulting slow accretion timescale means that heat can, in principle, be radiated efficiently from accreting bodies, meaning that differentiation can be avoided (Barr and Canup 2008). Third, the low surface densities mean that Type I migration (equation (3)) is slowed, making it more possible for growing satellites to survive. Numerical models show that, even though early generations of satellites may be lost in this scenario, outcomes resembling the Galilean satellites are common (Canup and Ward 2006; Ogihara and Ida 2012), and the overall process regulates the satellite system mass ratio to $\sim 10^{-4}M_{p}$, consistent with the observed mass ratios of the outer satellite systems (Canup and Ward 2006). Finally, slow inflow is also required so that Jupiter’s radius contracts to within Io’s orbit (Papaloizou and Nelson 2005), allowing Io to form (or once formed, survive).

The gas-starved disk model is undoubtedly simplified. For instance, it assumes a wide centrifugal radius $r_{c}$ (up to 30 $R_{J}$), a constant gas-to-solids ratio and a simple viscosity model. Nonetheless, it solves several fundamental problems posed by the MMSN in a framework that can also explain the Saturnian satellites (Canup and Ward 2006; Peale and Canup 2015), and has been correspondingly influential. A key question not addressed in the initial works on this concept (Canup and Ward 2002, 2006) was how satellitesimals large enough to accrete via mutual collisions into the Galilean satellites were able to grow within (or alternatively, be delivered to) the CPD. This topic, as well as the effect of different values of $r_{c}$ (and a decreting vs. an accreting disk in the satellite region), has been emphasized in subsequent models of inflow-supplied disks as described below.

#### Compositional Implications

The gas-starved disk model posits that the material within the disk, comprising both gas and solids, originates from the protosolar nebula (PSN). These solids likely included a variety of ices, organic matter, and refractory materials, as observed in comets and inferred for carbonaceous asteroids, both of which may act as proxies for the composition of the primordial building blocks of moons. The evolution of icelines within circumplanetary disks (CPDs) is driven by cooling processes, which are themselves influenced by the evolution of the infall rate (Fig. 1).

Several models of gas-starved disk evolution have been explored by Sasaki et al. (2010), who examined the impact of gap formation on the orbit of the host planet. Their results suggest that the evolution of the Jovian or Saturnian CPD unfolds in two distinct phases: the first corresponds to a steady-state flux of material delivered to the disk, while in the second, the infall rate abruptly decreases due to gap formation, effectively isolating the host planet from the PSN. The locations of the various icelines are determined by the equilibrium curves of the relevant condensates and are dependent on the evolution of the CPD’s infall rate. A classical explanation for the density dichotomy between the inner Galilean moons, Io and Europa, and the outer moons, Ganymede and Callisto, suggests that Io and Europa’s embryos formed inside the water iceline, while those of Ganymede and Callisto formed farther out (Canup and Ward 2002; Ronnet et al. 2017).

A notable feature of icelines is the *cold finger effect*, in which the surface density of icy solids increases at the iceline location due to the outward diffusion of vapors across the icelines, which are generated by the sublimation of inward-drifting ices (Stevenson and Lunine 1988; Anderson et al. 2021). This density bump increases the ice-to-rock ratio in solids near the iceline (or icelines). As a result, the ice fraction recorded by an accreting moon might be larger than the bulk ice fraction in materials delivered to the CPD. pressure bumps is correct, a Ganymede forming closer to the ice-line should be more ice-rich than Callisto. The opposite is the case (Table 1), implying that either this model is wrong or that other processes (e.g. tidally-driven volatile loss) have happened.

A major challenge for compositional models is that we do not know the starting materials. The surface compositions of the Galilean satellites are imperfectly known at present, and may have been so processed that they contain limited information on the starting materials (Sect. 3.4). A leading cosmochemical model posits that a gap in the PSN created as Jupiter grows in mass separates the accretion zones of non-carbonaceous and carbonaceous asteroids, inside and outside the gap respectively (Kruijer et al. 2020). Presumably, solids from both inside and outside of the gap could have fed Jupiter’s growing satellites, but the relative proportions of each are unknown. One possibility is that the materials from outside the gap were carbon-rich in the manner of comets (Ronnet et al. 2018; Reynard and Sotin 2023), but no known meterorite type has the carbon or organic matter content thought typical of comets (Bardyn et al. 2017).

A further possibility is that the satellite starting materials contained abundant phyllosilicates (clays). This hypothesis could explain Europa’s current ice content (Table 1) without requiring any icy material (Melwani Daswani et al. 2021). In order to explain Ganymede and Callisto’s icier composition, outwards diffusion of released water vapor would be required (Mousis et al. 2023). In this scenario, species more volatile than water are not accreted, which would make the Jovian satellites differ from, e.g., Enceladus.

The formation of the Galilean moons in a water-depleted CPD is also consistent with the supersolar abundance of heavy elements in Jupiter’s envelope (Sect. 2). These may have come from solids and/or vapors beyond the PSN snowline (Mousis et al. 2021; Aguichine et al. 2022), with the CPD forming when the planet migrated to a water-depleted region inside the snowline (Ali-Dib et al. 2014). Note that any such migration might result in disruption of the orbits of distant satellites (e.g., Namouni 2010).

### Decretion Disk

A relatively new idea in Galilean satellite formation is that of the decretion disk (Batygin and Morbidelli 2020). The models of Canup and Ward (2002, 2006) assume that satellites accrete in the region of the disk supplied by the inflow, i.e., interior to $r_{c}$. However, if the centrifugal radius is very small (perhaps only a few planetary radii), most of the CPD will experience outwards radial flow of gas and the satellites may accrete in this “decreting” region that is exterior to the region supplied by the inflow. Although sufficiently large bodies will still experience gas drag and net inwards migration, there will be a size range where the outwards motion of the gas balances the inwards drift due to gas drag. This balance allows solids to persist and the solids-to-gas ratio to increase at particular regions in the CPD (Drążkowska and Szulágyi 2018; Batygin and Morbidelli 2020). As the solids settle towards the mid-plane, local gravitational instabilities occur leading to the formation of satellitesimals. The subsequent accretion and migration of these satellitesimals is then similar to that outlined by Canup and Ward (2002, 2006). One difference is that a single region of satellitesimal formation exterior to the current satellites is envisioned that implies sequential moon formation, starting with Io and ending with Callisto, with each moon forming with a similar bulk composition and then migrating inward prior to the accretion of the next satellite.

In this scenario an inner disk cavity is required, and results in a primordial Laplace resonance forming from the inside out. Because of the high solid surface density, satellite accretion is fast (of order 1–10 kyr). Such rapid growth would lead to temperatures inconsistent with an undifferentiated Callisto (see Sect. 4.1.1). The authors suggest that Callisto formed later and more slowly, as a consequence of the disappearance of the CPD gas.

A decretion disk that allows efficient planetesimal formation beyond Callisto’s orbit may require a dynamical mechanism to later clear these leftover objects (Batygin and Morbidelli 2020). One possible mechanism is the dynamical instability of the solar system’s giant planets (Sect. 5.2); however, its impact on the evolution of the Galilean system remains unclear within this scenario.

Finally, an issue not discussed in the initial paper (Batygin and Morbidelli 2020) is that of bulk composition. wm Batygin and Morbidelli (2020) have the satellites form at a great distance from their present positions, in a cold nebular environment, which predicts that their “Io” would initially have been very ice-rich. However, it appears difficult to subsequently remove volatiles by tidal heating (Bierson and Steinbrügge 2021), in which case an initially ice-rich Io is hard to reconcile with its present-day state.

### Pebble Accretion

While some models consider that large satellitesimals may form efficiently from small solids within the CPD (e.g., Drążkowska and Szulágyi 2018; Batygin and Morbidelli 2020), this remains uncertain. If small CPD particles do not efficiently form satellitesimals within the CPD, an alternative solution is that large planetesimals formed within the proto-planetary disk (PPD) may be captured intact during passage through the CPD (Sect. 4.6), providing “seeds” for satellite growth. A key question is then how efficiently such bodies may grow into satellites via the sweep-up of the small CPD particles before the particles drift inward and are lost.

The nature of accretion of small particles by a much larger body–and how this depends on disk and pebble properties–is a process known as pebble accretion (Ormel and Klahr 2010; Lambrechts and Johansen 2012). Pebble accretion is widely used to explain the properties of the planets in the solar system and in exoplanet systems (Johansen and Lambrechts 2017; Liu and Ji 2020; Drążkowska et al. 2023). Pebble accretion relies on the concept that gas drag acts on particles during their encounter with a gravitating body dissipates energy to such an extent that the particle becomes trapped in the Hill radius of the planetary body. It will then settle to the surface of the body on a settling timescale. The original attraction of pebble accretion is that once a suitable “seed” is present, accretion can be extremely rapid. This solved the problem of Jupiter’s growth: conventional accretion models found it challenging to grow a ∼10 Earth mass body on which gas could accrete before the gas dissipated (see Sect. 2).

A few factors determine the effectiveness of the pebble accretion process:

1. The aerodynamical size or Stokes number $\tau _{s}$. This dimensionless parameter, the product of the stopping time and the orbital frequency, quantifies the impact of gas drag. Particles with $\tau _{s} \ll 1 $ are tightly coupled to the gas, while for particles with $\tau _{s} \gg 1$ gas only matters on secular timescales. Pebbles with $\tau _{s}\sim 1$ drift the fastest. Pebble accretion typically involves Stokes numbers in the range $10^{-3..-2} \lesssim \tau _{s} \lesssim 1$, though the precise limits are model dependent.
2. The pebble accretion onset mass $R_{\mathrm{init}}$. Pebble accretion only operates on bodies exceeding a critical radius $R_{\mathrm{init}}$ (Visser and Ormel 2016). In CPD research it is often supposed that planetesimals can be captured from the protosolar nebula, or protoplanetary disk (PPD) (Suetsugu and Ohtsuki 2017). However, planetesimals smaller than $R_{\mathrm{init}}$ can only accrete via their geometrical cross sections, which is highly inefficient. The onset radius depends on the aerodynamical size of the pebbles, with smaller pebbles allowing smaller seed bodies.
3. The pebble accretion efficiency ϵ, that is, the fraction of material that is accreted as opposed to drifting past. At high radial drift velocities, pebble accretion becomes inefficient as pebbles drift past the satellite’s orbital location without experiencing an encounter.

In Table 2 typical numbers for the aerodynamical sizes, the onset mass, and the pebble accretion efficiency are given in both the circumplanetary disks and the protoplanetary disk. In the CPD the dependence of $\tau _{s}$ on particle size is nonlinear due to the Stokes drag regime. But despite the nonlinearity, Stokes number are broadly comparable to those in the PPD. The onset radius for pebble accretion, $R_{\mathrm{init}}$, in the CPD amounts to diameters in the range $100{-}700\text{ km}$; these are the “seeds” from which the Galilean satellites could then grow. Capturing such large planetesimals can be challenging, particularly in the outer regions of the CPD (Ronnet and Johansen 2020; Suetsugu and Ohtsuki 2017). Depending on the tendency of the seeds to migrate inwards and be lost (see below) four or more seeds would be required for the Galilean system.

**Table 2:** Representative values for Stokes number, the pebble initiation size, and pebble accretion efficiencies (ϵ) for the environment of the protoplanetary disk (PPD) and the circumplanetary disk (CPD). Entries listed a(b) read $a{\times}10^{b}$. In calculating ϵ a diffusivity parameter of $\alpha _{z}=10^{-4}$ has been assumed for both the CPD and PPD. PA=pebble accretion

Environment		PPD (5.2 au)			CPD (15.3 R_*J*_)		
Surface density Σ	[g cm^−2^]	180			4 × 10^3^		
Gas aspect ratio *H*/*r*		0.05			0.1		
Particle size	[cm]	0.1	1	10	0.1	1	10
Stokes number $\tau _{s}$		1.4(−3)	0.014	0.14	2.1(−4)	0.018	0.24
PA onset radius $R_{init}$	[km]	140	270	520	46	160	330
Accretion efficiency *ϵ*	%						
– at 10 $m_{\oplus}$		22	23	11	3.6	13	4.4
– at 0.1 $m_{\oplus}$		0.2	0.7	0.6	0.04	0.3	0.4

In disks lacking pressure bumps, pebble accretion is generally inefficient. The pebble accretion efficiency is a highly sensitive to disk conditions, however, with cooler and more quiescent disks ensuring higher efficiencies. For small $\tau _{s}$ pebble accretion typically operates in the 3D limit, which is inefficient (Ormel 2017), but even at higher $\tau _{s}$, efficiencies are limited by the strong pebble drift. The pebble accretion efficiency ϵ is a useful metric, as it directly informs us of the total mass in pebbles that had flowed through the CPD: $M_{\mathrm{needed}} \sim m_{\mathrm{satellites}}/\epsilon $, where $m_{\mathrm{satellites}}$ is the total mass of the Galilean satellites. For an efficiency of 10%, this amounts to a total pebble mass of ${\sim}1\,m_{\oplus}$. And at the canonical solids-to-gas ratio of 1:100, it implies a mass 100 $m_{\oplus}$ in gas must have flowed through the CPD. Thus, if the satellites grow primarily via the accretion of small particles, there is little wiggle room to allow for further mass loss processes, such as satellite ingestion by the planet. This efficiency problem is a potentially significant issue for pebble accretion; it would, however, be alleviated in colder CPDs or when the gas flow is directed outwards (Szulágyi et al. 2022), or in general if satellitesimal formation within the CPD is efficient, so that satellite growth is dominated by satellitesimal-satellitesimal collisions rather than by pebble accretion.

In recent years, several studies have explored scenarios that explain the properties of the Galilean system with pebble accretion. Some constraints are not unique to the pebble accretion model, however. For example, satellite migration and subsequent loss of satellites would further exacerbate the solid mass budget problem detailed above. To retain the satellites, pebble accretion formation models (Shibaike et al. 2019; Ronnet and Johansen 2020; Madeira et al. 2021) invoke the existence of a disk truncation radius where Type I migration is either halted or is reversed (Sasaki et al. 2010). In such scenarios satellites would naturally pile up in mean-motion resonances. Addressing the mass budget issue, Ronnet and Johansen (2020) have pointed out that planetesimals captured from the PPD into the CPD would undergo strong thermal ablation, lifting the metallicity of the CPD. The ablated material then readily coagulates into pebbles.

Using a semi-analytical model Shibaike et al. (2019) replicated most observational properties of the Galilean system, including the satellite masses, composition, and dynamical characteristics. Their model suggests that the Galilean satellites formed slowly from pebbles, beginning with the injection of four “seed” bodies in the outer regions of the disk, followed by gradual accretion of pebbles. The low pebble densities and the correspondingly low opacity in the disk kept the disk sufficiently cold, promoting accretion of ices. In addition, the slow growth of the satellites mitigates ^26^Al-induced heating, which aligns with Callisto’s likely undifferentiated interior. In this scenario, the high rock fraction of the inner moons is explained by small pebbles losing ice by sublimation on crossing the snow-line. Accretion times in their models are, however, quite long, in excess of 10 Myr.

A subsequent N-body study by Madeira et al. (2021) focused more closely on the dynamical evolution of the system. They found that four initial seeds were unlikely to survive and instead favor capture of multiple seeds. From the ensuing dynamical shakeup Madeira et al. (2021) showed that a Galilean-analogue system could emerge, where all four planets reside in a 2:1 resonance chain. In their scenario the satellites exhibit an initially wetter and more uniform composition due to the giant-impact dynamics, and eccentricities that are far in excess of their present-day values. However, the authors contend that post-disk tidal processes (e.g., dynamical tides) would damp the eccentricities and let Callisto escape the 2:1 resonance (Lari et al. 2023). In addition, water from the inner satellites may have been removed during the accretion process due to sublimation of ice from pebbles (Wang et al. 2023) or through hydrodynamic escape of an early water ocean (Bierson and Nimmo 2020). Whether either process could have outcompeted radial mixing (Dwyer et al. 2013) and resulted in the monotonic density gradient observed remains to be seen.

Finally, a more radical concept is that satellite formation was delayed until after gas dispersal, and was sourced from a ring orbiting Jupiter. In this picture, solid material spreads outwards from the ring and satellites emerge beyond the Roche limit (Kokubo et al. 2000). Ring torques then drive the satellites further outwards (Crida and Charnoz 2012; Salmon and Canup 2012). While this mechanism may explain how the Saturnian (and potentially the Uranian) satellites formed, it does not appear to be responsible for the formation of Jupiter’s satellite system (Crida and Charnoz 2012).

### The Role of Heliocentric Impactors

The recognition that gas accretion onto proto-Jupiter was mostly vertical and derived from above the disk midplane suggested that the accreting gas might be depleted in solids (Shibaike et al. 2019; Ronnet and Johansen 2020; Batygin and Morbidelli 2020) (Sect. 2). While this topic is the subject of current debate (Szulágyi et al. 2022), the perceived problem of solids delivery has prompted some authors to consider heliocentric planetesimals as a potential source of material to form the Galilean satellites. An early investigation was by Estrada et al. (2009); more recent works include (Shibaike et al. 2019; Ronnet and Johansen 2020). Unfortunately, the details of this process are complicated and depend on poorly known parameters such as the size-distribution of the planetesimals (e.g. the number of potential “seeds”), the timing of giant planet migration, the effects of fragmentation and so on. Nonetheless, the inferred super-solar enhancement of heavy elements in Jupiter’s envelope (Sect. 2) is one possible indication that delivery of solids via heliocentric planetesimals was important.

## Early Post-Accretion Evolution

In an ideal world, one would be able to use present-day characteristics to directly infer the conditions under which the satellites formed. The problem is that many of the initial characteristics were *overprinted* and thus retain no information regarding accretion conditions. Io is so volcanically active that it has lost almost all information about earlier times, except perhaps in its sulphur (and other) isotopes. Here we will briefly discuss likely post-formation processes, and then examine what present-day characteristics might still be telling us about satellite origins.

For convenience we will discuss chemical/thermal evolution and dynamical evolution separately below. However, in reality one of the main energy sources driving thermal/chemical evolution is tidal heating, so the two topics are strongly coupled.

### Chemical/Thermal Evolution

The Galilean satellites will have experienced primordial heating due to accretion, by an amount that depends on the size-distribution of the impactors and the accretion timescale (Sect. 4.1.1). The conductive timescale for heat to diffuse out of a body of radius R is $R^{2}/\pi ^{2} \kappa $, where κ is the thermal diffusivity. Even for a small moon like Europa, this timescale is longer than the age of the Solar System, so some primordial heat may have been retained to the present day.

In addition to primordial heat, three further sources of energy are available to maintain the heat of these bodies over different timescales. The first is radioactive decay from the long-lived isotopes of K,U and Th contained in the silicate portions of the moons. Assuming chondritic abundances, the total energy release from these isotopes over 4.5 Gyr is equivalent to a silicate temperature increase of about 1000 K (Turcotte and Schubert 2002). Of course, this heat energy may be advected or diffused away, and the presence of ice will reduce the total heating rate. Thus, internal temperatures could in principle get high enough to melt rock or iron, but this is by no means assured. Conversely, melting of ice is hard to avoid unless heat transfer is efficient (e.g. the ice convects; Nagel et al. 2004).

The second energy source available is tidal heating. Sustained and substantial tidal heating due to eccentricity tides generally only arises if an orbital resonance (such as the Laplace resonance) is present. In this case, the damping of eccentricity due to dissipation in the moons can be balanced by the increase in eccentricity driven by the resonance. Ultimately, energy is being taken from the rotational kinetic energy of the primary and transferred to the satellites, where it is dissipated as heat. Thus, the total long-term tidal heating rate in the satellites depends on the rate at which the primary can supply heat (Lissauer et al. 1984; Meyer and Wisdom 2007; Peale and Canup 2015), and *not* on the satellite characteristics. This affords a very helpful simplification, particularly as astrometry can be used to infer a present-day dissipation rate in Jupiter (Lainey et al. 2009). On the other hand, inferring tidal heating in the past requires a knowledge of the positions of the satellites and what resonances they occupied, neither of which is well known.

How the tidal heating is partitioned *between* the satellites cannot be ascertained without knowledge of the satellites’ dissipative capacities. At the present day, Io dominates the tidal heating budget, but there may have been episodes in the past when Europa was more strongly heated than at present (Ojakangas and Stevenson 1986; Hussmann and Spohn 2004). The age of the Laplace resonance is unknown (see below); prior to capture into this resonance, other orbital configurations may have existed (Showman and Malhotra 1997). For instance, Ganymede may have experienced resurfacing due to passage through a “Laplace-like” resonance in the past (Showman et al. 1997) prior to it entering the current resonance. Callisto is not currently in any resonance; whether it has encountered resonances in the past is uncertain (Downey et al. 2020; Lari et al. 2023), but any tidal heating induced by such resonances would have to be consistent with its geologically pristine appearance.

One important observational constraint is the fact that sulphur and chlorine isotopes in Io’s thin atmosphere are extremely fractionated (de Kleer et al. 2024). Gravitational settling in the upper atmosphere results in preferential loss of the lighter isotopes. If these species go through multiple cycles of outgassing, loss and reincorporation into the interior, large isotopic anomalies can build up. Io appears to have lost >94% of its sulphur over geological time, indicating that it must have been at least as active as the present day over timescales comparable to the age of the solar system (de Kleer et al. 2024). This result favors an ancient origin of the Laplace resonance (or an earlier, equivalently heat-generating resonance).

Ultimately, sulphur loss at Io is being driven by tidal heating. However, calculations by Bierson and Steinbrügge (2021) suggest that tidally-driven water loss was not efficient at either Io or Europa (even substantial Io-like heating within modern Europa would not translate to efficient loss of water vapor to space). If so, the observed radial density gradient in ice fraction must be a consequence of accretion, and not later processes. This is a important conclusion.

The third source of energy is the thermal energy emitted by the surrounding circumplanetary disk. As discussed in Sect. 4.1, this disk is heated by several mechanisms, including accretion, viscous stress, the temperature of the surrounding protosolar nebula, and radiative heating from Jupiter, whose surface temperature can reach up to ≈2000 K due to accretional heating (Szulágyi et al. 2016). As Ganymede and Callisto are ice–rich, their accretion is typically assumed to have started beyond the snowline in the circumplanetary disk. As they migrated inwards, they would have accreted solids with temperatures reflecting the local conditions in the disk at the time.

If the Galilean satellites accreted relatively cold (see above), they may have initially consisted of an undifferentiated rock-ice mixture (as Callisto may be today). Subsequent warming by radioactive decay - potentially aided by tidal heating - would have driven subsequent differentiation. This would have taken place in two stages: first a separation of rocks from ice, as the ice melted; and, later, melting of silicates and Fe or Fe-S and the formation of a metallic core. An example of such a scenario applied to Europa may be found in Trinh et al. (2023), but it should be emphasized that the uncertainties in the initial conditions and the degree of tidal heating render such scenarios highly non-unique. In the absence of samples, how the timing of differentiation could be derived through observation is unclear.

### Dynamical Evolution

After accretion of the Galilean satellites, they will have experienced outward orbital migration due to tidal interaction with Jupiter. The orbital migration timescale is proportional to the 6.5 power of the Moon’s orbital semimajor axis (Goldreich and Soter 1966), and thus Io is primarily subject to tidal torque. When Io is in mean motion resonances with the outer satellites, the angular momentum received is efficiently distributed to Europa and Ganymede, causing the entire resonance chain to move outward (Yoder 1979; Yoder and Peale 1981). The actual extent of the outward migration depends on Jupiter’s tidal quality factor $Q_{\mathrm{J}}$, which has not been fully determined. Astrometric observations of the Galilean satellites have led to estimates that Jupiter’s tidal dissipation function $Q_{\mathrm{J}}$ is about $10^{5}$ at Io’s tidal frequency (Lainey et al. 2009). Measurements at Saturn however indicate that Q can be both low and frequency-dependent (Lainey et al. 2020); if a similar situation applies at Jupiter then the outwards evolution of some of the moons may have been more rapid than expected (Downey et al. 2020; Lari et al. 2023).

The timing of establishment of the Laplace resonance is an open question. Gas-driven inwards migration during the accretion epoch could have naturally led to the resonance (Sect. 3.2). But so too could later, Jupiter-driven outwards migration (Yoder and Peale 1981). The apparent episode of middle-aged heating on Ganymede suggests that resonances were encountered prior to the present one (Sect. 3.3), but this is uncertain. An important constraint pointed out by Batygin and Morbidelli (2020) is that Io and Europa had to be locked into a 2:1 resonance prior to establishment of the 2:1 Europa-Ganymede resonance. In contrast, Peale and Lee (2002) model the formation of the 2:1 Ganymede-Europa resonance as forming first in the CPD, driven by Ganymede’s strong Type I migration. The order in which the Laplace resonance was established has potentially profound implications for the initiation and magnitude of tidal heating within Io and Europa (Peale 2003; Batygin and Morbidelli 2020)).

N-body simulations conducted by Madeira et al. (2021) which account for planet migration and orbital damping within the circumplanetary disk reveal that establishing the Laplace resonance among the three innermost Galilean satellites is a non-trivial process. As the satellite precursors migrate inward toward the model’s assumed disk inner edge, set at approximately 5$R_{J}$, convergent migration frequently leads to dynamical scattering, collisions, and orbital rearrangements among the growing satellites. In about 10% of the simulations, the four innermost satellites form a long resonant chain, where all evolve in a 2:1 mean motion resonance. Their results suggest that the probability of any satellite pair being captured into a 2:1 mean motion resonance is approximately ∼30–50%.

If the Laplace resonance was established early, its survival may also provide a clue to Solar System dynamical evolution. It is widely accepted that the giant planets of the Solar System initially formed in a more compact orbital configuration and later evolved into their current dynamical state due to a planetary dynamical instability (Gomes et al. 2005; Levison et al. 2011; Nesvorný and Morbidelli 2012). Numerical simulations show that during this phase Jupiter, Saturn, Uranus, and Neptune experienced substantial orbital evolution and may have also undergone close encounters with one another. Notably, some scenarios suggest that Jupiter may have experienced a close encounter with an additional ice giant, leading to a rapid change in Jupiter’s orbit (Nesvorný and Morbidelli 2012). One of the most intriguing consequences of such an event is its potential impact on Jupiter’s Galilean moons. Deienno et al. (2014) investigated how planetary encounters during this period of instability might have influenced the orbits of Io, Europa, Ganymede, and Callisto. Their simulations revealed that sufficiently close encounters between Jupiter and another giant planet could have significantly perturbed the orbits of Jupiter’s major satellites. These interactions could have led to increased eccentricities, modifications to Callisto’s semi-major axis, and, in some cases, the disruption of Laplace resonance (if it existed at that time). Their findings also indicate that the inclinations induced by the encounters’ gravitational perturbations would likely have persisted today. This last conclusion neglects possible damping by obliquity tides, which can be important for ocean-bearing worlds (Downey et al. 2020).

The simulations by Deienno et al. (2014) were based on the assumption of a relatively late planetary instability. However, recent cosmochemical and dynamical studies suggest that this instability may have occurred much earlier, taking place no later than ∼100 million years after the Solar System’s formation (Morbidelli et al. 2018; Nesvorný et al. 2018; de Sousa Ribeiro et al. 2020), and potentially as early as ∼5–10 million years (Liu et al. 2022). If the Solar System’s instability coincided with the dispersal of the protoplanetary gas disk, it is possible that Jupiter still retained an active circumplanetary disk at that time. In such a scenario, a close encounter between Jupiter and an extra ice giant could have had profound (and largely unexplored) consequences shaping the structure and long-term evolution of Jupiter’s circumplanetary disk and the final formation and orbital architecture of Jupiter’s moons.

Jupiter’s other satellites may also bear witness to the formation and evolution of the Galilean satellites. Although knowledge of the satellites interior to Io is sparse, the mass of the largest satellite, Amalthea, was determined by a close Galileo flyby (Anderson et al. 2005) The derived density of 860±100 kg m^−3^ was interpreted by these authors as implying Amalthea’s interior contains a substantial fraction of water ice. If this is true, and not withstanding whether Amalthea and the other inner moons are leftovers from the CPD or birthed from a more massive primordial ring (as described above), the implication is that migration of the ice line, satellitesimal drift, or dynamical mixing provided ice interior to Io’s accretion zone. It therefore seems almost inescapable that Io accreted with at least some water or ice in its makeup. Amalthea is also interesting because its orbit has been explained as the result of an initial inwards migration of Io (Brunton and Batygin 2025), placing constraints on early CPD characteristics.

At the other edge of the Galilean satellite system, it is notable that there are no regular midsize icy satellites outside Callisto’s orbit, in contrast to the presence of both Hyperion and Iapetus outside the orbit of Titan. Perhaps the CPD around Saturn had different characteristics to that around Jupiter? Oberg et al. (2020) offer that strong FUV flux into the jovian gap from the young Sun’s stelllar birth cluster truncated the protojovian CPD. This may have prevented the formation of outer regular satellites, and possibly slowed the inward migration of Callisto, preventing it from entering into resonance with the other Galilean satellites. Analogous effects are not seen at the Saturn system, however.

Finally, there are the distant irregular satellites. Although now viewed as captured bodies from the primordial Kuiper belt (Lowry et al. 2008), they are relevant to the Galilean satellites. Given the long geologic history expressed on the major moons, it is doubtful that any direct remnants of the primordial rocky solids that built these worlds remain on their surfaces. The exception of course is Callisto. But the question is whether the dark, presumably carbon-rich material that blankets Callisto (Moore et al. 2004) is representative of Callisto’s original satellitesimal building blocks, or is, rather, debris accumulated from the early collisional evolution of the irregulars (Bottke et al. 2013). Future compositional measurements and comparisons may settle the issue, and possibly provide a direct indication of the composition of the solids accreted into the protojovian CPD.

### Are There Surviving Primordial Characteristics?

Whether or not we can use present-day characteristics of the Galilean satellites to infer their mode of formation depends on whether these characteristics have been overprinted by later processes. Here we will briefly summarize the most salient points.

In terms of physical characteristics, the two most important are the bulk composition and the differentiation state. Bulk composition is hard to modify by post-formation processes (Bierson and Steinbrügge 2021). The observed density gradient is therefore a consequence of the time- and spatially-variable conditions in the disk, and must have survived the effects of radial mixing and migration. The details of how this works in practice are unclear, and depend on the size of the accreting materials. Most (but not all) of the accretion scenarios discussed above are able to reproduce the observed gradient, so its usefulness as a discriminant is limited.

Ice-rock differentiation during accretion is hard to avoid, and requires long accretion timescales and an absence of large impactors (Sect. 4.1.1). Later ice-rock differentiation due to radioactive decay and/or tidal heating could potentially have been avoided because of convection. If Callisto is not fully differentiated, then that places tight constraints on acceptable accretion scenarios and its subsequent evolution. Unfortunately, as explained in Sect. 3.1, whether or not Callisto is fully differentiated is based on the so-far unverified assumption of hydrostatic equilibrium.

Orbital characteristics provide limited information. Except (perhaps) for Callisto, satellite eccentricities are not primordial but instead reflect the Laplace resonance (or earlier resonances). The existence of the Laplace resonance itself is surely a clue, but we do not know its age, nor how it was established (e.g., outwards vs. inwards migration). Ganymede’s middle-age tidal heating event hints at a pre-existing resonance, while Io’s strong sulphur isotope signal suggests persistent, long-term tidal heating.

The surface chemistries of the Galilean satellites may contain information about initial disk characteristics, but so much subsequent processing (both endogenic and exogenic) has probably occurred that their utility is currently limited. Isotopes are more hopeful because they are less subject to later modification. In particular, measured D/H and carbon isotopic ratios may be telling us something about the provenance of the material from which the Galilean satellites formed (Sect. 3.4). However, isotopic loss (as at Io or potentially Europa; Sect. 5.1) may have overprinted the original signals. Even so, measuring D/H at Europa, Ganymede and Callisto may retain value as a constraint.

## Outstanding Questions

The Galilean satellites contain clues, however fragmentary, to their mode of formation and the environment in which they formed. Based on the analysis above, below we pose a series of questions regarding the satellites’ origin and evolution and briefly describe their importance. We have organized the questions in rough chronological order.

**1. How did gas accrete to the circumplanetary disk, and did it develop an inner cavity?** The details of gas delivery determine whether the disk is accretionary or decretionary, while if a disk cavity develops (which depends on the disk and planet magnetic fields) then that limits or even prevents inwards migration of growing satellites (Sect. 4.1).

**2. How, when, and in what form were solids delivered to the disk?** The solids-to-gas ratio in the infalling material is currently under dispute (Sect. 2), and the solid size-frequency distribution is unknown, but both have strong implications for satellite growth processes. For instance, if much of the solid mass was delivered by heliocentric objects, that would radically change the picture of accretion. Further, it remains unclear where and when small solids in a CPD might have been sufficiently concentrated to form large satellitesimal “seeds” via local gravitational collapse.

**3. What was the thermodynamical and chemical evolution of the disk?** Local conditions (Sect. 4.1) will have set the initial rock-ice ratio, and any subsequent evolution must have somehow preserved the observed radial gradient in ice fraction. Was the disk affected by external factors such as the protosun’s stellar birth environment, or a close encounter with another giant planet?

**4. What solid material did the moons accrete, and how fast did accretion happen?** The size of the accreting material and the rate of accretion both control the thermal effects of accretion (Sect. 4.1.1). The composition of the material will determine the satellite bulk composition, unless accretion-driven volatile loss occurs. Can the non-ice components of the moons be directly related to known meteorite types, or to cometary compositions? And were ices more volatile than water ice accreted, especially to Ganymede and Callisto? Solids of all scales probably underwent radial migration (Sect. 4.1), complicating the overall picture.

**5. When did the Laplace resonance begin, and were there pre-existing resonances?** The Laplace resonance could have been established by either inwards or outwards migration of the satellites (Sect. 5.2). As a result, when it was established remains an open question. If Ganymede’s resurfacing was a consequence of an earlier resonance (Sect. 3.2), this implies that the Laplace resonance was established relatively late and does not place any constraint on early dynamical evolution of the Solar System. How did Callisto escape entering into a mean motion resonance with the other satellites, or if it was once in resonance, how did it break free?

**6. What is the current rate of satellite migration?** Ultimately, the engine driving geological activity in the Galilean satellites is dissipation in Jupiter. Since this quantity controls the rate of satellite migration (Sect. 5.2), a measurement of migration rates provides important constraints on the system as a whole. In particular, the history of resonances depends on the relative migration rates of the satellites.

## How Well Will Juno, JUICE, Clipper and Tianwen-4 Address These Questions?

At least some of the questions posed above will be answered, in part or in whole, by Juno and the upcoming JUICE, Europa Clipper and Tianwen-4 measurements. In our view, the questions most likely to be answered by these missions are as follows:

**6. What is the current rate of satellite migration?** Both JUICE and Clipper will make flybys of Callisto, and the spacecraft will then focus on Ganymede and Europa, respectively, while Tianwen-4 will orbit Callisto. Given the expected ∼10 cm/yr migration rates, the accumulated along-track displacement relative to the no-migration case will be of order 10 km since *Galileo*. Except for Io, future spacecraft ephemeris determinations will be several orders of magnitude more precise than this (e.g., Magnanini et al. 2024). Further analysis of Juno data may provide an improved estimate of Io’s migration rate. These measurements will provide data on Jupiter’s dissipation in a manner analogous to that obtained at Saturn (Lainey et al. 2020).

**4. What solid material did the moons accrete, and how fast did accretion happen?** The 21 planned JUICE flybys of Callisto will determine Callisto’s MoI (and thus its differentiation state) (Cappuccio et al. 2022). A Callisto orbiter, such as Tianwen-4, would yield additional geodetic information (e.g. rotation state) (Huang et al. 2025). If Callisto is only partially differentiated, that would indicate slow accretion from small bodies (Sect. 4.1.1). Gravity studies, though non-unique may be able to determine the size of Europa’s core (if any), potentially providing a further constraint on accretion. Chemical or isotopic measurements, either of sputtered or impact ejected surface materials or plumes (if present), could help provide crude constraints on the solids. For instance, D/H, carbon or oxygen isotope ratios could be interpreted in terms of the original provenance of the material (Sect. 3.4), potentially modulated by loss processes (Sect. 4.1.1).

**3. What was the thermodynamical and chemical evolution of the disk?** Compositional and isotopic measurements (see above) could provide information on provenance (e.g. where did the gas originate?) and process (e.g., what species condensed at different distances?).

## What High-Priority Questions Will Not Be Answered, and What Are the Key Future Measurements?

**1. How did gas accrete to the circumplanetary disk, and did it develop an inner cavity?** It seems unlikely that truly definitive answers to this question will be obtained by studying the Galilean satellites. A more likely source of answers is observations of extant CPDs around other stars. Current observations by JWST and ALMA are just capable of resolving such disks (e.g., Benisty et al. 2021; Blakely et al. 2025) and will doubtless improve with time.

**2. How, when, and in what form were solids delivered to the disk?** Although spacecraft measurements of isotope ratios may provide some insight (see above), their precision is limited. It is now thought that solar system materials come in two subtly different (few parts per million) isotopic flavors: “carbonaceous”, presumed to be from the outer solar system beyond Jupiter; and “non-carbonaceous”, inwards of Jupiter (Sect. 4.3.1.) (Kruijer et al. 2020). Accordingly, a sufficiently precise measurements of the Galilean satellites’ isotopic ratios for elements like Ti, Cr, Zn, Fe,W, Mo might be very illuminating. For instance, it would likely reveal the extent to which Jupiter really did interrupt inwards migration of solids. Such precise measurements would require samples to be analyzed in terrestrial laboratories. This is not totally out of the question: Io erupts materials far into space where they could, in principle, be collected by a spacecraft and returned to Earth (Ogliore et al. 2024).

**5. When did the Laplace resonance begin, and were there pre-existing resonances?** This again seems like a hard question to answer. Further investigation of Ganymede’s apparent resurfacing event might yield some insight. One could anticipate future isotopic measurements and models for Io’s loss processes improving our understanding of how long tidal heating has been operating (Sect. 5.1). But it seems difficult to determine whether this heating was due to the Laplace resonance or some earlier resonance.

## Summary and Conclusions

The study of how the Galilean satellites formed has benefited from new observations (from Galileo, Juno and Earth-based observations) and also new theories.

On the observational side, there is a possibility (yet to be confirmed) that Callisto is undifferentiated or only partially differentiated (Sect. 3.1); if so, this places strong constraints on how it could have formed (Sect. 4.1.1). The steady density progression of the Galilean satellites (Sect. 3.1) has been long-known but remains a fundamental observation. Ganymede apparently underwent a resurfacing event mid-way through its history (Sect. 3.3); if so, this implies the Laplace resonance was not primordial. Astrometric measurements provide some constraints on how the satellites may have migrated outwards over geological time (Sect. 5.2). And isotopic measurements hint that Io has been geologically active over most of its history (Sect. 5.1).

On the theory side, the old idea of a minimum-mass subnebula (Sect. 4.2) has been largely superseded. Instead, the starved-disk model (Sect. 4.3) is influential because it yields colder temperatures and relatively slow accretion timescales compared with the traditional minimum-mass subnebula (Lunine and Stevenson 1982). Alternative developments include the decretion disk model (Sect. 4.4) and pebble accretion (Sect. 4.5). Unresolved questions of importance include the issue of whether the disk developed an inner cavity (Sect. 2), the efficiency with which solids were delivered to the CPD, and their source (did they co-accrete with gas, or were they from a separate reservoir?). Although the observed density gradient is a first-order constraint, not all accretion models successfully reproduce it.

A general difficulty with inferring accretion processes from present-day observations is that of overprinting (Sect. 5.3). Many processes, notably tidal heating and radiogenic heating, may have caused zeroth-order changes to internal structure and composition long after the satellites formed. One exception to this, however, is bulk composition — with the exception of Io, it seems very likely that the ice-rock ratio of these bodies was established by the end of accretion and has not changed since. Isotopic measurements (e.g. D/H) should contain information about primordial provenance, but may also have been affected by escape. Orbital characteristics are unlikely to be primordial, except perhaps for Callisto. Overall, Callisto is the body which has been least re-set by subsequent processing, hence its particular importance as a tracer of accretional processes.

We anticipate that many of the questions posed here (Sect. 6) will be answered by the JUICE, Europa Clipper and Tianwen-4 missions (Sect. 7). Some, however, are likely to prove more intractable (Sect. 8). Whether new missions will be selected in the foreseeable future to answer them is unclear. More likely, improvements in theory and, especially, observations of protoplanetary and even circumplanetary disks in other star systems are likely to provide the breakthroughs.

## Footnotes

1 The concern has diminished in recognition that the length of time for this stage is sensitive to opacity and other factors and can readily be compatible with the suspected longevity of the nebula and meteoritic evidence, roughly 5 Myr.

2 It is clearly not solid to begin with since it is driven to supercritical temperatures, of order $10^{5} K$ during accretion, but it might solidify at a later stage since isentropic compression of liquid silicates, for example, may cause solidification and the much later stages of Jupiter evolution may tend towards isentropic conditions. This is not known at present.

3 The size needed to encompass 15 Earth masses of purely heavy elements (keeping in mind the high density of silicate and iron at high pressure).

4 A small nearly pure heavy element inner core is not excluded by the data and preferred in some models.
